# Inhibition of mTORC1 signaling protects kidney from irradiation-induced toxicity via accelerating recovery of renal stem-like cells

**DOI:** 10.1186/s13287-018-0963-5

**Published:** 2018-08-14

**Authors:** Lijian Shao, Wuping Yang, Rui Xu, Shuqin Zhu, Yanqiu Huang, Huan Li, Xincheng Wu, Mengzhen Yue, Xiaoliang Xiong, Xiaowen Chen, Bohai Kuang, Guangqin Fan, Qingxian Zhu, Huihong Zeng

**Affiliations:** 1 Jiangxi Provincial Key Laboratory of Preventive Medicine, Nanchang University, Nanchang, 330006 China; 2 School of Public Health, Nanchang University, Nanchang, 330006 China; 3 Department of Histology and Embryology, Basic Medical School of Nanchang University, No. 461 Bayi Road, Nanchang, 330006 China; 4 Department of Pathology, The Fifth Affiliated Hospital of Sun Yat-sen University, Zhuhai, 519000 China; 5 The First Affiliated Hospital of Nanchang University, Nanchang, 330006 China; 6 Department of Pathology, Basic Medical School of Nanchang University, Nanchang, 330006 China; 7 Department of Medical Genetics and Cell Biology, Basic Medical School of Nanchang University, Nanchang, 330006 China

**Keywords:** Irradiation, Renal damage, mTOR complex 1, Rapamycin, Apoptosis

## Abstract

**Background:**

Irradiation-induced kidney damage is inevitable during radiotherapeutic practice, which limits effective radiotherapy doses on tumor treatment. In the present study, the role of mTOR complex 1 (mTORC1) signaling was investigated in irradiation-induced renal injuries.

**Methods:**

Mice were exposed to 8.0-Gy X-ray of total body irradiation and subsequently treated with rapamycin. Changes of renal morphology were assessed by hematoxylin and eosin staining. Expression of pS6 and CD133 was detected via immunostaining. Cellular apoptosis and proliferation were measured by TUNEL, caspase-3 and BrdU staining. Activation of mTORC1, TGF-β and NF-κB signaling pathways was determined through western blot analysis.

**Results:**

Our data displayed that irradiation disrupted the structures of renal corpuscles and tubules and decreased the density of CD133^+^ renal stem-like cells, which were related with increasing cellular apoptosis and decreasing cell proliferation post exposure. Activation of mTORC1, TGF-β and NF-κB signaling pathways was determined in irradiated renal tissues, which were inhibited by rapamycin treatment. Application of rapamycin after irradiation decreased cellular apoptosis and increased autophagy and cell proliferation in renal tissues. The density of CD133^+^ renal stem-like cells was significantly increased in irradiated kidneys after rapamycin treatment. The morphology of irradiated renal corpuscles and tubules was gradually recovered upon rapamycin treatment.

**Conclusions:**

These findings indicate that inhibition of mTORC1 signaling by rapamycin ameliorates irradiation-induced renal toxicity mediated by decreasing cellular apoptosis and increasing CD133^+^ renal stem-like cells.

**Electronic supplementary material:**

The online version of this article (10.1186/s13287-018-0963-5) contains supplementary material, which is available to authorized users.

## Background

Radiotherapy is often considered an effective approach for treating tumors in the upper abdomen, including the pancreas, stomach, liver and esophagus [1, 2]. However, effective radiation doses on tumors are limited by the damage to normal tissues. Irradiation-induced kidney dysfunction seems inevitable when patients experience radiotherapy treatment, such as total body irradiation before bone marrow transplantation and radionuclide therapy, and so forth [3, 4]. The reasons for radiotherapy-induced acute neuropathology are mainly ascribed to irradiation-induced cell death at renal glomerular and tubular levels, such as apoptosis, autophagic cell death and necrosis [5, 6]. Irradiation-induced DNA damage is also an important carcinogen to the kidney [7]. However, a countermeasure to irradiation-induced kidney dysfunction is currently unavailable, which might be due to missing detailed investigation of its underlying mechanisms.

Although it is well known that hematopoietic and intestinal systems are very sensitive to irradiation, the kidney is a radiosensitive organ as well [8, 9]. For example, exposure to 28 gray (Gy) of radiation to both kidneys in 5-week-old mice will result in renal failure [10]. The risk of renal impairment will further increase when combined with chemotherapy [10]. Acute and chronic kidney dysfunction can be induced by irradiation. In irradiation-induced acute kidney damage, abnormal histological changes are easily identified at renal glomerular and tubular levels whereas kidneys look normal in size and shape through B ultrasound and MRI examinations. Therefore, irradiation-induced acute nephrotoxicity can easily be ignored in clinical diagnosis. The chronic clinical manifestations of kidney dysfunction usually appear 1–1.5 years or longer after radiotherapy treatment, which is characterized by an increase in serum creatinine, proteinuria, anemia and hypertension [11]. In the treatment of Wilms tumor for 108 patients, 73% of patients, receiving a dose of irradiation > 23 Gy, had an increased level of serum creatinine [12]. It has been documented that more than 40% of patients, who received 8–12 Gy of total body irradiation, developed a reduced estimated glomerular filtration rate in a 5-year follow up [13]. The mechanisms of irradiation-induced chronic kidney damage are not well known, which might be related to the activation of different signaling pathways including the nuclear factor kappaB (NF-κB) and transforming growth factor beta (TGF-β) pathways [14, 15]. Another possibility is that acute damage might be one of the mediators in irradiation-induced chronic kidney injury.

The primary factor of irradiation-induced acute damage is cell death through apoptosis, autophagy and necrosis. Previous studies have shown that irradiation could quickly induce apoptosis in hematopoietic and intestinal stem cells, which could be inhibited by deletion of puma gene [16, 17]. Exposure of 8-Gy irradiation to rats significantly increased cellular apoptosis and reactive oxygen species (ROS) production in the kidneys. Antioxidant quercetin treatment could decrease irradiation-induced apoptosis and ROS production in the kidneys [18]. Activation of autophagy has dual roles, either pro or anti cell death. Modulation of the mTOR complex 1 (mTORC1) signaling pathway can efficiently regulate autophagy activation. Rapamycin inhibits mTORC1 signaling, leading to activation of autophagy [19]. However, several questions about mTORC1 signaling remain elusive under irradiation conditions: whether mTORC1 signaling is acutely activated in irradiated kidneys; and whether inhibiting mTORC1 signaling protects the kidneys from irradiation-induced damage.

To test these possibilities, mice were exposed to an 8-Gy dose of irradiation and mTORC1 signaling was examined. Our results showed that irradiation activated not only NF-κB and TGF-β signaling but also mTORC1 signaling. Importantly, inhibition of mTORC1 signaling by rapamycin treatment alleviated renal morphological damage and increased the numbers of kidney stem-like cells upon irradiation, which might be mediated by decreasing cellular apoptosis and increasing autophagy in irradiated kidneys.

## Methods

### Animals and irradiation

Eight-week-old male C57BL/6 J mice (*n* = 50) were purchased from Hunan SLAC Laboratory Animal Co., Ltd (Certificate Number: SYXK 2011–0003) and shipped to Nanchang University. After a 1-week acclimation period, mice received X-ray total body irradiation (8.0 Gy, 2.28 Gy/min, Elekta Precise accelerators). Sham-treated animals underwent the same procedures as the irradiated groups but received no irradiation (*n* = 5 mice per group). The mice were housed under a constant 12-h light:dark cycle. Food and water were provided ad libitum. Animals were analyzed at days 1, 3 and 7 after irradiation (*n* = 5 mice per time point). All procedures were approved by the Institutional Animal Care and Use Committee at Nanchang University.

### Rapamycin treatment

Rapamycin (> 98% purity) was purchased from Dalian Meilun Biotechnology Co. Ltd (Cas:53123-88-9; China). Rapamycin was dissolved in ethanol at 10 mg/ml and diluted in 5% Tween-80 and 5% polyethylene glycol 400 (Solarbio Science & Technology Co., Ltd, China). For rapamycin treatment, 6 h after irradiation, mice were treated with rapamycin (4 mg/kg) by subcutaneous (s.c.) injection and then the injection was repeated every other day. As a control, mice were irradiated or nonirradiated and received s.c. injection of the same volume of vehicle (200 μl).

### Histopathological and immunohistochemical examination

The renal tissues were fixed in 4% paraformaldehyde and then embedded in paraffin and cut into 5.0 μm sections. Sections were used to perform hematoxylin and eosin (HE) staining for histological examination and to measure expression of phosphorylated S6 and cleaved caspase-3 by immunohistochemistry staining. Briefly, after dewaxing, dehydration, rehydration and antigen retrieval with microwave, paraffin sections were blocked with 3% H_2_O_2_ deionized water and subsequently incubated with the specific primary antibody against phosphorylated S6 (1:600; Cell Signaling Technology, USA) and cleaved caspase-3 (1:1000; Cell Signaling Technology, USA) at 4 °C overnight, followed by staining with horseradish peroxidase-conjugated secondary antibody. The substrate diaminobenzidine (DAB) was used for coloration. Immunostained sections were counterstained with hematoxylin to visualize the nuclei and examined under an optical microscope (Olympus, Japan).

To analyze the integrated optical density (IOD) of pS6 and cleaved caspase-3, five visual fields (per immunohistochemical slice) were randomly selected under high magnification (10 × 40) and photographed. The IOD of pS6 and cleaved caspase-3 in kidneys was calculated via the HMIAS-2000 image analysis system with high resolution and multicolor.

For cell proliferation assay, 5-bromo-2′-deoxyuridine (BrdU, 100 mg/kg; Beijing Solarbio technology Co. Ltd, China) was intraperitoneally injected 2 h before mice were euthanized. Immunohistochemistry was used to detect BrdU-positive cells in kidneys. BrdU-positive cells were counted under a light microscope and presented as percentages of BrdU-positive cells in each field.

### TUNEL assay

To detect the fragmented nuclear DNA associated with apoptosis, a standard terminal deoxynucleotidyl transferase (TdT)-mediated deoxyuridine triphosphate (dUTP)-biotin nick-end labeling (TUNEL) method was employed on paraffin sections. For this purpose, the in-situ cell apoptosis detection kit I, POD (Boster, China) was used according to the manufacturer’s instructions. Briefly, renal tissues were fixed in 4% paraformaldehyde and embedded with paraffin. After standard deparaffinization, hydration and incubation with 3% hydrogen peroxide at room temperature for 10 min and proteinase K at 37 °C for 10 min, tissue sections were incubated: with labeling buffer, TdT and DIG-dUTP (19:1:1) at 37 °C for 2 h; with blocking reagent at room temperature for 30 min; with biotin antidigoxin antibody at 37 °C for 30 min; and with SABC at 37 °C for 30 min. Diaminobenzidine was used as the chromogen. For physiologically positive controls, sections of mouse small intestine were subjected to the same procedure. For negative controls, some slides were incubated with label solution that did not contain TdT. The number of TUNEL-positive cells was counted from five randomly selected fields at 400× magnification per kidney sample.

### Western blot analysis

For western blotting, the renal tissues post irradiation were frozen in liquid nitrogen until further use. Protein extraction was carried out using the RIPA buffer (Applygen, Beijing, China). The BCA protein assay Kit (Applygen) was used to quantitate total protein levels. Protein (40 μg per lane) was separated by SDS-PAGE. All proteins were separated on 10% gel. Proteins were transblotted to PVDF membranes in standard Tris–glycine transfer buffer, pH 8.3, containing 0.1% SDS. After transfer, membranes were blocked for 3 h at room temperature in TBST (10 mmol/L Tris–HCl, pH 8.0, 150 mmol/L NaCl, 0.1% Tween-20) containing 5% nonfat milk powder or containing 5% BSA and incubated overnight at 4 °C with anti-S6 (1:1000; Cell Signaling Technology, USA), anti-phospho-S6 (1:2000; Cell Signaling Technology, USA), anti-p65 (1:1000; Abcam, UK), anti-phospho-p65 (1:1000; Abcam, UK), anti-Smad3 (1:1000; Abcam, UK), anti-phospho-Smad3 (1:1000; Abcam, UK), anti-LC3-I/II (1:500; Affinity, USA), anti-mTOR (1:1000; Abcam, UK), anti-phospho-mTOR (S2448, 1:1000; Abcam, UK), anti-Akt (1:5000; Abcam, UK), and anti-phospho-Akt (S473, 1:5000; Abcam, UK) diluted in TBST containing 5% nonfat milk powder or 5% BSA. Membranes were then washed in TBST for 30 min, incubated with horseradish-peroxidase-conjugated goat anti-rabbit IgG, diluted 1:10,000 (Beijing Zhongshan, China) in TBST containing 5% nonfat milk powder or 5% BSA, washed in TBST for 30 min and resolved by chemiluminescence (Beijing TIANDZ, China). All membranes were stripped and reprobed with anti-GAPDH antibody (Proteintech, China) as a loading control. The band densities from western blot analysis were quantitated using ImageJ software (https://imagej.nih.gov/ij/).

### Statistical analysis

All parameters were expressed as the mean ± standard deviation. The data were analyzed by analysis of variance (ANOVA). Differences among group means were analyzed by Student–Newman–Keuls multiple comparisons test after one-way or two-way ANOVA. Differences were considered significant at *p* < 0.05. All analyses were done with GraphPad Prism from GraphPad Software.

## Results

### Irradiation-induced acute kidney injury

**Fig. 1:**
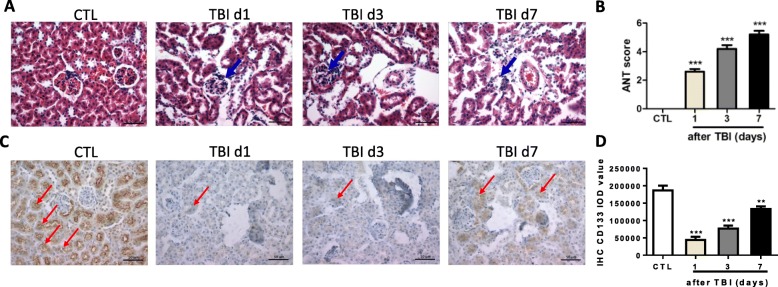
Irradiation-induced renal toxicity in mice. C57BL/6 J mice exposed to 8.0 Gy of total body irradiation (TBI). Kidney tissues harvested at days 1, 3 and 7 (d1, d3, d7) post exposure. **a** Representative pictures by HE staining. Arrows represent damaged corpuscles. **b** Acute tubular necrosis (ANT) score in nonirradiated and irradiated kidneys. **c** Immunostaining of CD133 in nonirradiated and irradiated kidneys. Arrows represent CD133-positive staining. **d** Integrated optical density (IOD) of CD133 staining presented via imaging analysis. Nonirradiated renal tissues (CTL) used as controls. Bar = 50 μm. **p < 0.01 vs CTL; ***p < 0.001 vs CTL

To investigate the effects of irradiation on the kidney, mice were exposed to 8 Gy of TBI and the histological changes of renal structure were examined at days 1, 3 and 7 post exposure. As shown in Fig. 1a, b, the kidneys in nonirradiated mice showed greater details of the renal tubules and corpuscles. Many tubules (proximal and distal) that reside adjacent to renal corpuscles contain a single layer of cuboidal epithelium. The corpuscles have Bowman’s capsule and glomerulus. The Bowman’s capsule has squamous epithelium at the urinary pole. The squamous epithelium of the partial layer switches to cuboidal epithelium of the proximal convoluted tubules. The glomerulus is a tuft of capillaries formed from the glomerular arterioles and surrounded by connective tissues. In the irradiated mice, the convoluted tubules showed obscure structures with partial or complete obstruction of its lumens. The cuboidal epithelium cells of proximal and distal renal tubules showed nuclear changes, mainly pyknosis and karyolysis with infiltrating leukocytes. Atrophied glomeruli were detected in the renal cortex. Bowman’s space became wider and the basement membrane appeared thicker in irradiated mice than those in nonirradiated mice.

It has been reported that adult stem-like cells exist in kidneys from different species, including mouse, rat and human [20, 21]. Label-retaining cells (LRCs) also accumulate in renal epithelial tubular cells in rat and mouse [22, 23]. CD133, one of cell surface markers, was used to identify renal stem-like cells in mouse, rat and human kidneys [20]. We therefore examined CD133 expression via immunostaining in kidneys before and after irradiation. As shown in Fig. 1c, d, the majority of CD133-positive immunostaining distributed in the renal tubules in unirradiated kidneys. Few CD133-positive cells were also detected in renal corpuscles, which is consistent with the previous publication [24]. However, the density of renal CD133 immunostaining was significantly decreased at day 3 after irradiation. By day 7 post exposure, the density of renal CD133 immunostaining had a trend to increase while it was much lower density than that in nonirradiated kidneys. These data imply that total body irradiation might damage stem-like cells in kidneys.

**Fig. 2:**
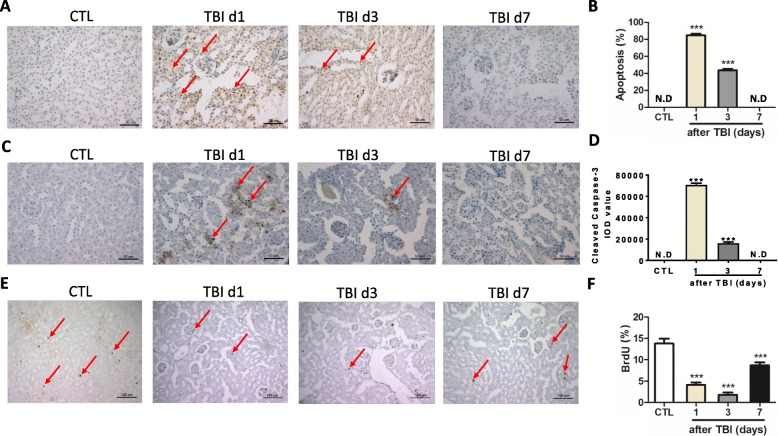
Irradiation-induced cellular apoptosis in kidney tissues. **a, b** Irradiated kidney tissues collected at days 1, 3 and 7 (d1, d3, d7) after total body irradiation (TBI). TUNEL staining used to detect cellular apoptosis. Representative pictures by TUNEL staining (**a**). Bar = 50 μm. Ratio of apoptotic cells (arrowed) in each field (**b**). **c, d** Irradiated kidney tissues collected at days 1, 3 and 7 after TBI. Cellular cleaved caspase-3 determined by immunostaining. Representative pictures by cleaved caspase-3 staining (**c**). Bar = 50 μm. Imaging software used to analyze integrated optical density (IOD) of cleaved caspase-3 (**d**). **e, f** BrdU incorporation assay in kidney tissues. BrdU (100 mg/kg) intraperitoneally injected 2 h before mice were euthanized. BrdU-positive cells (**e**, arrowed) counted under light microscope and percentages of BrdU-positive cells in each field presented (**f**). Bar = 100 μm. ***p < 0.001 vs CTL. CTL nonirradiated renal tissues, N.D nondetected

Irradiation-induced tissue damage is often mediated by the induction of cellular apoptosis. TUNEL staining was thus used to measure the ratio of apoptosis in irradiated kidneys (Fig. 2a, b). Rare apoptotic cells can be observed in nonirradiated kidneys. However, nearly 80% and 40% of cells in the kidneys were stained positive with TUNEL at days 1 and 3 after irradiation, respectively. By day 7 post exposure, most of the apoptotic cells were cleaned out. Fragmented DNA demonstrated by TUNEL staining can appear in both apoptotic and necrotic cells. To double-check irradiation-induced cellular apoptosis in the kidneys, cleaved caspase-3 antibody was used to stain apoptotic cells (Fig. 2c). Our data showed that the density of caspase-3-positive staining was significantly increased at day 1 after irradiation compared to that in nonirradiated mice (Fig. 2d). This is consistent with the data from TUNEL staining even though there was less cleaved caspase-3-positive staining than TUNEL-positive staining at day 1 after irradiation. In addition, we also measured percentages of proliferating cells in irradiated kidneys by BrdU incorporation assay. As shown in Fig. 2e, f, around 14% of renal cells were in the S phase in nonirradiated kidneys. The percentages of proliferating cells were decreased to nearly 4% and 2% at days 1 and 3 after irradiation, respectively. By day 7 post exposure, the proliferating cells were gradually increased to around 7%. Collectively, these results indicate that irradiation causes renal damage including stem-like cell injury, cellular apoptosis and cell cycle arrest.

### mTORC1 signaling was activated in irradiated kidneys

It has been documented that irradiation affected multiple pathways, such as TGF-β and NF-κB signaling [14, 15]. Smad3 is an important component in the TGF-β signaling pathway. Upon activation of TGF-β signaling, Smad3 will be phosphorylated to form complex with Smad4 and activate downstream targets. As shown in Additional file 1: Figure S1a, b, minor pSmad3 could be detected in normal kidneys, while the levels of pSmad3 were initially increased at day 1 after irradiation and up to the peak level at day 3. The levels of pSmad3 were dropped back to normal levels at day 7 after irradiation. Activation of TGF-β signaling may be involved in irradiation-induced chronic tissue fibrosis. Similarly, NF-κB signaling in the irradiated kidney was activated, which was supported by the increased levels of phosphorylated p65 in a time-dependent manner after irradiation (Additional file 1: Figure S1c, d). Activation of NF-κB signaling may be related to irradiation-induced inflammation along with tissue damage.

**Fig. 3:**
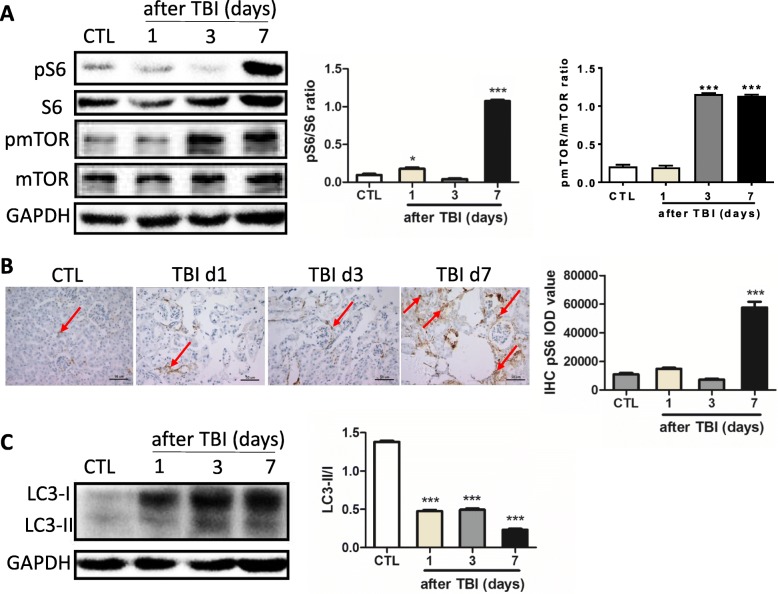
mTORC1 signaling activated by irradiation in kidney tissues. **a** Expression of pS6 and pmTOR upregulated post irradiation in kidney tissues. Protein lysates prepared from kidney tissues at days 1, 3 and 7 (d1, d3, d7) after irradiation. Expression of pS6, S6, pmTOR and mTOR (left panel) detected by western blotting. GAPDH used as housekeeping control. Nonirradiated kidney tissues (CTL) used as controls. Expression of pS6, S6, pmTOR and mTOR (right panel) quantitated by ImageJ software and ratios of pS6/S6 and pmTOR/mTOR presented. **b** pS6 immunostaining in kidney tissues. Kidney tissues fixed in 4% paraformaldehyde after irradiation and pS6 immunostaining performed as indicated in Methods (left panel). Bar = 50 μm. Imaging software used to analyze integrated optical density (IOD) of pS6 (right panel). Arrows represent pS6-positive staining. **c** Expression of LC3-I and LC3-II in kidney tissues. Protein lysates prepared from kidney tissues at days 1, 3 and 7 after irradiation. Expression of LC3-I and LC3-II (left panel) detected by western blotting. GAPDH used as a housekeeping control. Nonirradiated kidney tissues (CTL) used as controls. Expression of LC3-I and LC3-II quantitated by ImageJ software and ratio of LC3-II and LC3-I expression presented (right panel). *p < 0.05 vs CTL; ***p < 0.001 vs CTL. CTL nonirradiated renal tissues, GAPDH glyceraldehyde 3-phosphate dehydrogenase, IHC immunohistochemistry, pmTOR pshosphorylated mammalian target of rapamycin, TBI total body irradiation

Irradiation stress can negatively affect energy metabolism, microenvironment and protein translation, and so forth [25]. The mTORC1 signaling pathway plays critical roles in sensing the changes of nutrition/energy and protein translation. Irradiation might affect the mTORC1 signaling pathway. To test this possibility, the levels of phosphorylated ribosomal protein S6 (pS6), a downstream target of the mTORC1 signaling pathway, were measured in irradiated kidneys. As shown in Fig. 3a, the levels of pS6 were moderately increased at day 1 post exposure when compared to nonirradiated kidneys. The levels of pS6 were significantly increased up to five-fold at day 7 post exposure when compared to nonirradiated kidneys. Activation of mTOR signaling was also supported by the expression of pmTOR, showing that the levels of pmTOR were increased by seven-fold at days 3 and 7 after irradiation when compared to nonirradiated kidneys (Fig. 3a). To further confirm the activation of mTORC1 signaling, immunostaining was used to demonstrate the density and distribution of pS6, showing that the density of pS6 staining was significantly increased at day 7 after irradiation compared with that in nonirradiated kidneys (Fig. 3b). pS6-positive staining in nonirradiated kidneys was mainly distributed in glomerulus and Bowman’s capsule (Fig. 3b). There was less pS6-positive staining in proximal and distal tubules in the irradiated kidneys compared to nonirradiated ones.

It is well known that inhibition of mTORC1 signaling results in the increasing activity of autophagy. We showed that mTORC1 signaling was activated after irradiation in Fig. 3a, b. To explore whether the activity of autophagy was affected upon irradiation condition, the expression of LC3-II/LC3-I was measured by western blot analysis, demonstrating that the ratio of LC3-II/LC3-I was significantly reduced in all irradiated groups when compared to nonirradiated controls (Fig. 3c). These data suggest that irradiation activates mTORC1 signaling in the kidneys, leading to inhibition of cellular autophagy.

### Rapamycin treatment attenuated irradiation-induced kidney injury

**Fig. 4:**
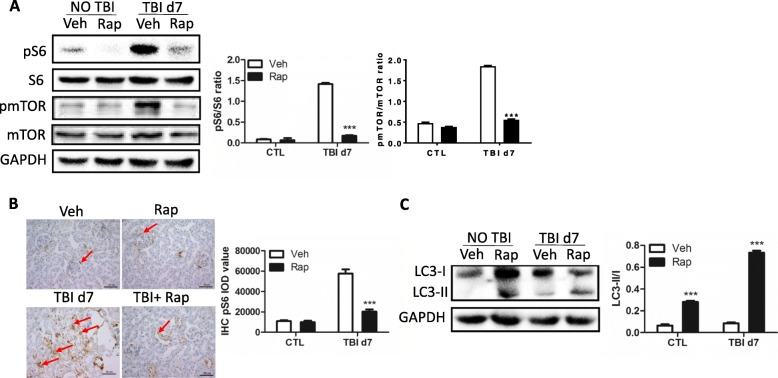
Rapamycin treatment reduced increase of pS6 expression in irradiated kidney tissues. **a** Increased expression of pS6 and pmTOR by irradiation reduced via rapamycin treatment. Protein lysates prepared from kidney tissues with (Rap) or without (Veh) rapamycin treatment at day 7 (d7) after irradiation. Expression of pS6, S6, pmTOR and mTOR (left panel) detected by western blotting. GAPDH used as a housekeeping control. Nonirradiated kidney tissues (CTL) used as controls. Expression of pS6 and S6 quantitated by ImageJ software and ratios of pS6/S6 and pmTOR/mTOR presented (right panel). **b** pS6 immunostaining in kidney tissues. Kidney tissues with or without rapamycin treatment harvested at day 7 post irradiation and fixed in 4% paraformaldehyde after irradiation and pS6 immunostaining performed as indicated in Methods (left panel). Bar = 50 μm. Imaging software used to analyze integrated optical density (IOD) of pS6 (right panel). Arrows represent pS6-positive staining. **c** Expression of LC3-I and LC3-II in kidney tissues. Protein lysates were prepared from kidney tissues at day 7 after irradiation. Expression of LC3-I and LC3-II (left panel) detected by western blotting. GAPDH used as housekeeping control. Vehicle treated-kidney tissues (Veh) used as controls. Expression of LC3-I and LC3-II quantitated by ImageJ software and ratio of LC3-II and LC3-I expression presented (right panel). *p < 0.05 vs Veh; ***p < 0.001 vs Veh. CTL nonirradiated renal tissues, GAPDH glyceraldehyde 3-phosphate dehydrogenase, IHC immunohistochemistry, pmTOR pshosphorylated mammalian target of rapamycin, TBI total body irradiation

Considering that irradiation activates mTORC1 signaling, the question is whether inhibition of mTORC1 signaling protects kidneys from irradiation-induced damage. Irradiated mice were thus treated with rapamycin, an inhibitor of mTORC1 signaling. As shown in Fig. 4a, the expression of pS6 in irradiated kidneys was significantly increased compared to nonirradiated controls. The increased levels of pS6 in irradiated kidneys were significantly reduced by rapamycin treatment. The increased levels of pmTOR induced by irradiation were significantly decreased by rapamycin treatment (Fig. 4a). It was confirmed that rapamycin treatment reduced the increased expression of pS6 in irradiated kidneys by immunostaining as shown in Fig. 4b. Furthermore, rapamycin treatment not only increased the ratio of LC3-II/LC3-I in nonirradiated mice but also raised the reduction of the LC3-II/LC3-I ratio in irradiated mice (Fig. 4c). These data indicate that rapamycin treatment inhibits the activation of mTORC1 signaling induced by irradiation and escalates cellular autophagy.

**Fig. 5:**
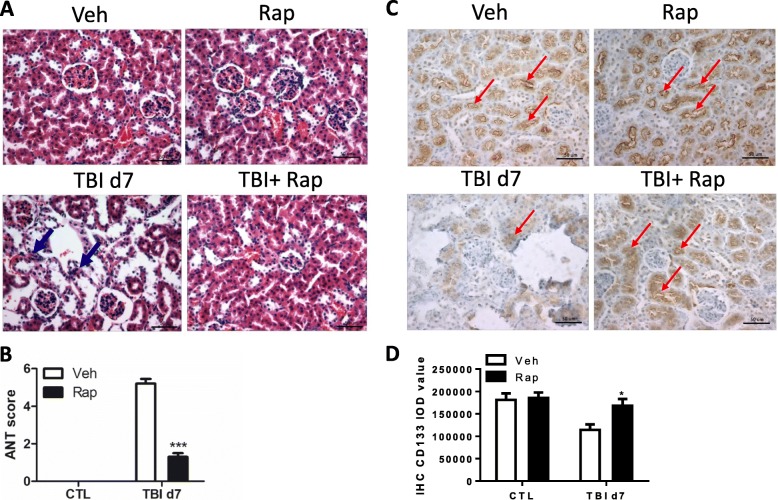
Rapamycin treatment attenuated kidney toxicity induced by irradiation. C57BL/6 J mice exposed to 8.0 Gy of total body irradiation (TBI) and treated with vehicle (Veh) or rapamycin (Rap) starting at 6 h post exposure. Mice treated with rapamycin every other day. Kidney tissues harvested at day 7 (d7) post exposure. **a** Representative pictures by HE staining. Arrows represent damaged corpuscles. **b** Acute tubular necrosis (ANT) score in kidneys with or without rapamycin treatment under irradiation conditions. **c** Immunostaining of CD133 in kidneys with or without rapamycin treatment under irradiation conditions. Arrows represent CD133-positive staining. **d** Integrated optical density (IOD) of CD133 staining presented via imaging analysis. Bar = 50 μm. *p < 0.05 vs Veh; ***p < 0.001 vs Veh. CTL nonirradiated renal tissues, IHC immunohistochemistry

To examine whether inhibition of mTORC1 signaling can protect the kidney against irradiation-induced injury, irradiated kidneys were analyzed at day 7 after irradiation under rapamycin treatment. As shown in Fig. 5a, rapamycin treatment did not affect the normal morphology in nonirradiated kidneys. However, irradiation induced significant morphological changes, including atrophied glomeruli, widened Bowman’s space and so on, which were ameliorated by rapamycin treatment. The acute tubular necrosis (ANT) scores of kidneys from irradiated mice which received rapamycin treatment were significantly declined compared with those of mice receiving irradiation only (Fig. 5b).

As shown in Fig. 1c, the expression of CD133, a renal stem-like cell marker, was disrupted after irradiation. To examine whether rapamycin treatment can accelerate the recovery of irradiation-damaged CD133^+^ cells, we observed irradiated kidneys at day 7 after irradiation with and without rapamycin settings. The results showed that rapamycin did not affect the expression of CD133 in nonirradiated kidney (Fig. 5c). Notably, CD133 expression was significantly increased in irradiated mice with rapamycin treatment when compared to those irradiated mice with vehicle treatment (Fig. 5c, d). These findings indicate that rapamycin treatment ameliorates abnormal renal morphology under irradiation condition, which might be due to promoting the recovery of renal stem-like cells upon rapamycin treatment.

**Fig. 6:**
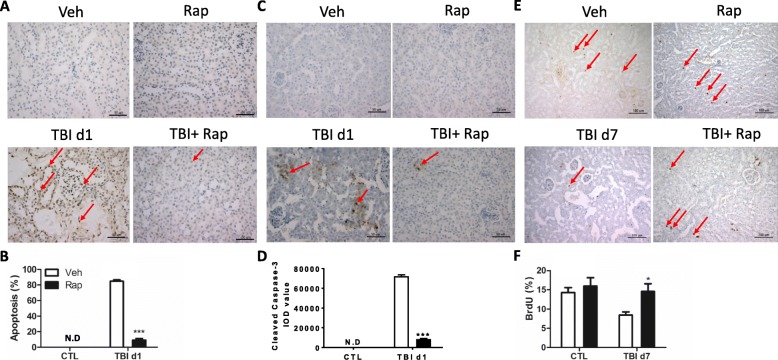
Rapamycin treatment decreased cellular apoptosis induced by irradiation. C57BL/6 J mice exposed to 8.0 Gy of total body irradiation (TBI) and treated with vehicle (Veh) or rapamycin (Rap) starting at 6 h post exposure. Kidney tissues harvested at day 1 (d1) or day 7 (d7) post exposure. **a, b** Cellular apoptosis induced by irradiation. Representative pictures by TUNEL staining (**a**, arrowed). Ratios of apoptotic cells in each field counted and presented (**b**). **c, d** Cellular cleaved caspase-3 determined by immunostaining at day 1 after irradiation. Representative pictures by cleaved caspase-3 staining (**c**). Imaging software used to analyze integrated optical density (IOD) of cleaved caspase-3 (**d**). (**e, f**). BrdU incorporation assay in kidney tissues. BrdU (100 mg/kg) intraperitoneally injected 2 h before mice euthanized. BrdU-positive cells (**e**, arrowed) counted under light microscope and percentages of BrdU-positive cells in each field presented (**f**). Bar = 100 μm. *p < 0.05 vs Veh; ***p < 0.001 vs Veh. BrdU 5-bromo-2′-deoxyuridine, CTL nonirradiated renal tissues, N.D nondetected

The protection of rapamycin treatment on irradiated kidneys might be involved in decreasing cellular apoptosis and increasing cell proliferation. Through TUNEL staining, rapamycin did not induce cellular apoptosis in nonirradiated kidneys, while the increased cellular apoptosis in irradiated kidneys was significantly reduced by rapamycin treatment (Fig. 6a, b). This is also supported by the data from cleaved caspase-3 immunostaining (Fig. 6c, d), showing that rapamycin treatment decreased the density of caspase-3-positive staining induced by irradiation. Rapamycin treatment did not change the ratio of BrdU incorporation in nonirradiated mice. Irradiation decreased renal cell proliferation by BrdU incorporation assay, which was partially rescued by rapamycin treatment (Fig. 6e, f). Rapamycin treatment also affected the changes of TGF-β and NF-κB signaling under irradiation conditions. As shown in Additional file 2: Figure S2a, b, rapamycin moderately increased the expression of pSmad3 in nonirradiated mice. The increased pSmad3 expression in irradiated kidneys was mildly decreased by rapamycin treatment. Additionally, rapamycin moderately dropped the levels of phosphorylated p65 expression in nonirradiated kidneys, while the increased expression of phosphorylated p65 by irradiation was completely abated via rapamycin treatment (Additional file 2: Figure S2c, d).

In addition, it is well known that activating mTORC2 signaling by S6 leads to Akt phosphorylation (S473). We thus measured expression of pAkt (S473) with and without rapamycin treatment under irradiation (Additional file 3: Figure S3). Our data showed that irradiation increased expression of pAkt at days 3 and 7 post irradiation in kidney (Additional file 3: Figure S3a, b). However, there was an increased trend in renal pAkt expression under irradiation and rapamycin treatment without statistical significance (Additional file 3: Figure S3c, d). Collectively, these findings suggest that rapamycin keeps the kidneys from irradiation-induced damage mediated by promoting the recovery of renal stem-like cells, decreasing apoptosis, inhibiting overactivation of TGF-β and NF-κB signaling induced by irradiation.

## Discussion

In the present study, we demonstrated that irradiation increased renal cell apoptosis and decreased cell proliferation and expression of CD133 in renal stem-like cells, leading to acute kidney injuries. These negative effects induced by irradiation might be related to the abnormal activation of mTORC1, TGF-β and NF-κB signaling pathways. Rapamycin treatment indeed ameliorated irradiation-induced vicious effects, which were proved by decreasing renal cell apoptosis and increasing cell proliferation and the levels of CD133 expression in renal stem-like cells, along with inhibiting activation of mTORC1, TGF-β and NF-κB signaling pathways under irradiation conditions.

Previous studies have shown that moderate single doses of irradiation caused renal damage in different species. For example, exposure of autologous bone marrow transplantation patients to 7.5 Gy of total body irradiation (TBI), even lower to 4.0 Gy, resulted in a significant decrease of the mean glomerular filtration rate and increase of serum creatinine and serum urea [26, 27]. Rats experienced a dose of 10 Gy of TBI, which will lead to acute and chronic radiation nephropathy [28, 29]. Exposure of dogs to 3.0 Gy of TBI will give rise to chronic renal damage including fibrosis [30]. Consistently, mice in the current study were exposed to 8.0-Gy X-ray TBI to generate acute kidney injury. The 8.0-Gy exposure in mice is approximately equivalent to 30 Gy for humans, which is basically equal to that for clinical use [31]. Our data demonstrated that 8.0-Gy TBI markedly induced renal damage in corpuscles and tubules and decreased the expression of CD133 in renal stem-like cells.

Previous studies have shown that irradiation induced cell death, at least in part, mediated by induction of cellular apoptosis, necroptosis, autophagy and DNA damage [32]. Our results showed that irradiation caused cellular apoptosis and slowed down cell proliferation, which might contribute to the decreased numbers of CD133^+^ stem-like cells and accelerating renal damage. Multiple signaling pathways are involved in cell proliferation and apoptosis [14, 15]. In the present study, we mainly examined three different pathways: mTORC1, TGF-β and NF-κB signaling. Extensive studies have worked on TGF-β and NF-κB signaling under irradiation settings [14, 15]. Expression of TGF-β1 receptor was significantly increased in acute and chronic renal toxicity after irradiation [33]. Acute activation of TGF-β1 signaling might be involved in kidney damage and related to irradiation-induced cellular apoptosis [34]. TGF-β1 signaling also anticipates irradiation-induced multiple organ fibrosis, such as kidney, lung and intestine. Inhibition of TGF-β1 signaling ameliorates irradiation-induced organ fibrosis by decreasing expression of collagen I and II [35, 36]. Recent data indicate that NF-κB signaling is activated in acute renal injuries post irradiation, which is related to irradiation-induced inflammation and apoptosis. Inhibition of NF-κB signaling can modulate irradiation-induced damage, such as decreasing expression of IL-6 and IL-8 and inhibiting mitogen-activated protein kinases [37, 38]. It is well known that autophagy can regulate both cell survival and death under different cell context situations. Activation of mTORC1 signaling inhibits cellular autophagy and is related to the activation of TGF-β and NF-κB signaling.

mTORC1 signaling can sense environmental amino acids, ATP, growth factors and insulin levels, and plays an important role in the regulation of cell growth and proliferation [39, 40]. Kinase mechanistic mTORC1 (target of rapamycin) plays an essential role in protein synthesis and is a central regulator of autophagy, which stimulates cell proliferation and represses autophagy when conditions are optimal for growth [41, 42]. In the present study, we demonstrated that irradiation significantly elevated the levels of pS6 and pmTOR post exposure when compared to the nonirradiated mice. These data imply that irradiation activates the mTORC1 signaling pathway in the kidney, which might modulate autophagy. The ratio of LC3-II/LC3-I expression was significantly decreased in irradiated renal tissues when compared to normal tissues, which is consistent with a previous theory that activation of the mTORC1 pathway is thought to be a negative regulator of autophagy [19].

Autophagy is associated with cell survival, cell death, inflammation and other pathological conditions. It can degrade cytoplasmic materials including cell organelles and plays a crucial role in maintaining intracellular homeostasis and cell survival [43]. It modulates cell survival under stresses, such as nutrient starvation, oxidative stress, hypoxia and DNA damage [44]. Our current data indicate that irradiation decreases the ratio of LC3-II/LC3-I expression at different time points after exposure. Combining irradiation-induced activation of mTORC1 signaling with inhibition of autophagy, mTORC1 signaling might be attributed to the irradiation-induced renal toxicity. Therefore, we hypothesize that inhibiting mTORC1 signaling results in activation of autophagy, leading to removal of dead cells induced by irradiation. This will play an important role in maintaining kidney intracellular homeostasis under pathologic conditions, such as DNA damage, oxidative stress and inflammation [45].

We utilized rapamycin, an acute specific inhibitor of mTORC1, to inhibit the activation of mTORC1 induced by total body irradiation. Our data demonstrated that rapamycin not only efficiently blocked the activation of mTORC1 signaling but also increased the ratio of LC3-II/LC3-I expression post irradiation, promoting the autophagy process, which will benefit removal of apoptotic and unrepaired DNA damaged cells. Beneficial effects of rapamycin treatment after irradiation are also supported by morphological observations. For example, rapamycin treatment decreased cellular apoptosis and increased cell proliferation along with accelerating the recovery of damaged renal glomerulus, Bowman’s capsule and tubules post exposure. The density of CD133^+^ renal stem-like cells in the irradiated kidney was significantly increased upon rapamycin treatment, which might play an important role during the recovery of damaged renal tissues. Furthermore, rapamycin treatment blocked the activation of TGF-β and NF-κB signaling pathways induced by irradiation, which might also contribute to the decreased cellular apoptosis and increased cell proliferation after rapamycin treatment. In addition, our data showed that rapamycin treatment had minor effects on the expression of mTORC2 target phosphorylated Akt (S473), indicating that inhibition of mTORC1 signaling plays a dominant role in the protection of rapamycin treatment under irradiation. Recently, long-term effects of rapamycin on irradiation-induced lung fibrosis have been reported, showing that rapamycin treatment attenuated lung fibrosis induced by irradiation [46]. It has been documented that quercetin had an ability to ameliorate mice kidney fibrosis mediated by inhibiting the mTOR signaling pathway [47]. In the irradiated salivary gland, inhibition of mTORC1 by rapamycin could prevent irradiation-induced salivary hypofunction in swine [48]. The radioprotection of rapamycin was also observed in human submandibular gland cell line [48]. The long-term effects of rapamycin treatment on the irradiated kidney remain warranted in future studies.

## Conclusion

In summary, our present study provides important findings that inhibiting mTORC1 signaling by rapamycin treatment benefits the recovery of injured kidneys after irradiation, which is mediated by decreasing cellular apoptosis, increasing autophagy and CD133^+^ renal stem-like cells, blocking activation of TGF-β and NF-κB signaling. These results may have some implications for treating acute kidney damage under stress conditions. Knowledge gained from the current study could also aid in planning countermeasure strategies to protect against negative effects of radiation exposure during radiotherapy and nuclear accidents.

## Additional files

Additional file 1: **Figure S1.** TGF-β and NF-κB signaling activated by irradiation in kidney tissues. (**A, B**) Expression of pSmad3 upregulated post irradiation in kidney tissues. Protein lysates prepared from kidney tissues at days 1, 3 and 7 after irradiation. Expression of pSmad3 and Smad3 (**A**) detected by western blotting. GAPDH used as a housekeeping control. Nonirradiated kidney tissues (CTL) used as controls. Expression of pSmad3 and Smad3 quantitated by ImageJ software and ratio of pSmad3/Smad3 presented (**B**). (**C, D**) Expression of pNF-κBp65 upregulated post irradiation in kidney tissues. Protein lysates prepared from kidney tissues at days 1, 3 and 7 after irradiation. Expression of pNF-κBp65 and NF-κBp65 (**C**) detected by western blotting. GAPDH used as a housekeeping control. Nonirradiated kidney tissues (CTL) used as controls. Expression of pNF-κBp65 and NF-κBp65 quantitated by ImageJ software and ratio of pNF-κBp65/NF-κBp65 presented (**D**). **p < 0.01 vs CTL; ***p < 0.001 vs CTL. (PDF 168 kb)

Additional file 2: **Figure S2.** Rapamycin treatment inhibited activation of TGF-β and NF-κB signaling induced by irradiation. C57BL/6 J mice exposed to 8.0 Gy of total body irradiation (TBI) and treated with vehicle (Veh) or rapamycin (Rap) starting at 6 h post exposure. Kidney tissues harvested at day 3 or day 7 post exposure to assess activation of TGF-β and NF-κB signaling, respectively. (**A, B**) Protein lysates prepared from kidney tissues at day 3 after irradiation with or without rapamycin treatment. Expression of pSmad3 and Smad3 (**A**) detected by western blotting. GAPDH used as a housekeeping control. Vehicle treated-kidney tissues (Veh) used as controls. Expression of pSmad3 and Smad3 quantitated by ImageJ software and ratio of pSmad3/Smad3 presented (**B**). (**C, D**) Protein lysates prepared from kidney tissues at day 7 after irradiation. Expression of pNF-κBp65 and NF-κBp65 (**C**) detected by western blotting. GAPDH used as a housekeeping control. Vehicle treated-kidney tissues (Veh) used as controls. Expression of pNF-κBp65 and NF-κBp65 quantitated by ImageJ software and ratio of pNF-κBp65/NF-κBp65 presented (**D**). ***p < 0.001 vs Veh. (PDF 163 kb)

Additional file 3: **Figure S3.** Levels of pAkt increased by irradiation. C57BL/6 J mice exposed to 8.0 Gy of total body irradiation (TBI) and treated with vehicle (Veh) or rapamycin (Rap) starting at 6 h post exposure. Kidney tissues harvested at days 1, 3 and 7 post exposure to assess levels of phosphorylated Akt (pAkt). (**A, B**) Expression of pAkt upregulated post irradiation in kidney tissues. Protein lysates prepared from kidney tissues at days 1, 3 and 7 after irradiation. Expression of pAkt and Akt (**A**) detected by western blotting. GAPDH used as a housekeeping control. Nonirradiated kidney tissues (CTL) used as controls. Expression of pAkt and Akt quantitated by ImageJ software and ratio of pAkt/Akt presented (**B**). (**C, D**) Protein lysates prepared from kidney tissues at day 7 after irradiation. Expression of pAkt and Akt (**C**) detected by western blotting. GAPDH used as housekeeping control. Vehicle treated-kidney tissues (Veh) used as controls. Expression of pAkt and Akt quantitated by ImageJ software and ratio of pAkt/Akt presented (**D**). ***p < 0.001 vs Veh. (PDF 91 kb)

## Abbreviations

ANOVA: Analysis of variance
ANT: Acute tubular necrosis
BrdU: 5-Bromo-2′-deoxyuridine
Gy: Gray
HE: Hematoxylin and eosin
IOD: Integrated optical density
LRC: Label-retaining cell
mTORC1: mTOR complex 1
NF-κB: Nuclear factor kappaB
pS6: Phosphorylated ribosomal protein S6
ROS: Reactive oxygen species
S6: Ribosomal protein S6
TBI: Total body irradiation
TBST: 10 mmol/L Tris–HCl, pH 8.0, 150 mmol/L NaCl, 0.1% Tween-20
TGF-β: Transforming growth factor beta
TUNEL: Terminal deoxynucleotidyl transferase dUTP nick end labeling
