# Supplementary figures and images for: Inhibition of mTORC1 signaling protects kidney from irradiation-induced toxicity via accelerating recovery of renal stem-like cells
Source: Stem Cell Res Ther. 2018 Aug 14;9:219. doi: 10.1186/s13287-018-0963-5 (PMC6092808; doi:10.1186/s13287-018-0963-5)

Figure S1

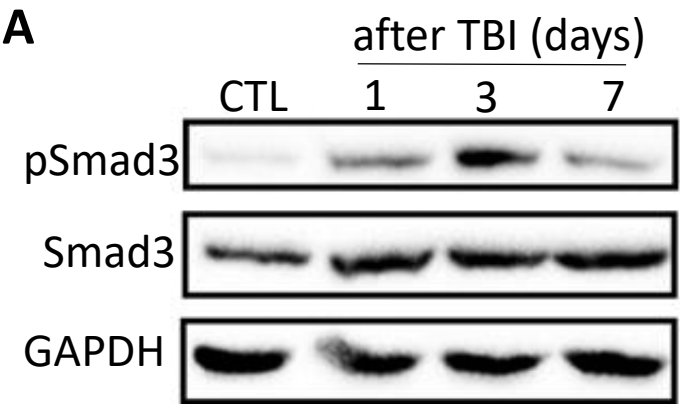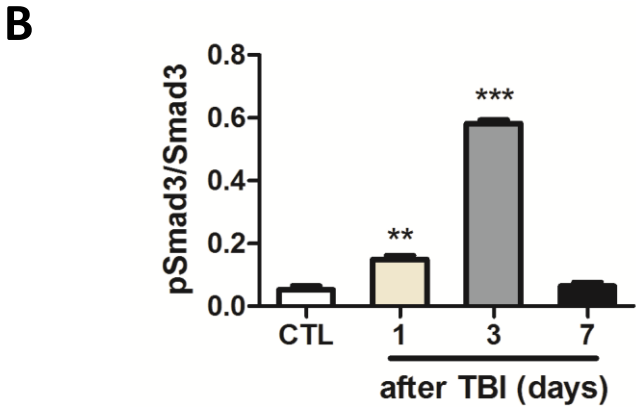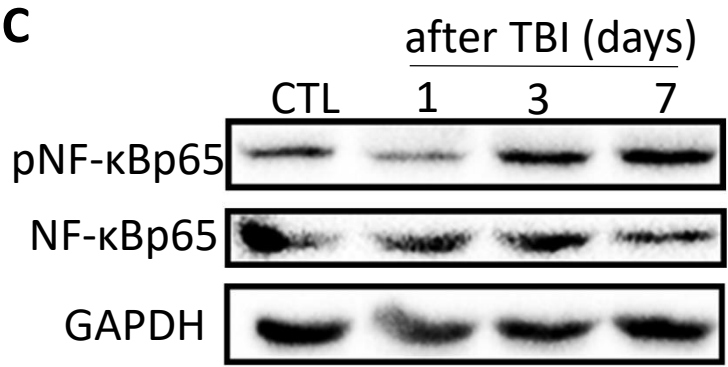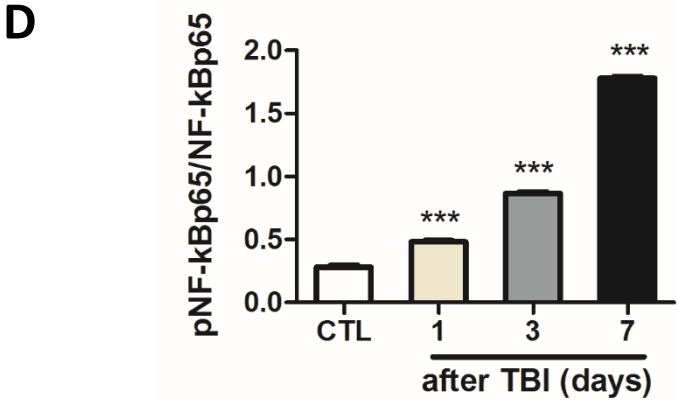

Supplement: Supplementary file 1 — Figure S1. TGF-β and NF-κB signaling activated by irradiation in kidney tissues. (A, B) Expression of pSmad3 upregulated post irradiation in kidney tissues. Protein lysates prepared from kidney tissues at days 1, 3 and 7 after irradiation. Expression of pSmad3 and Smad3 (A) detected by western blotting. GAPDH used as a housekeeping control. Nonirradiated kidney tissues (CTL) used as controls. Expression of pSmad3 and Smad3 quantitated by ImageJ software and ratio of pSmad3/Smad3 presented (B). (C, D) Expression of pNF-κBp65 upregulated post irradiation in kidney tissues. Protein lysates prepared from kidney tissues at days 1, 3 and 7 after irradiation. Expression of pNF-κBp65 and NF-κBp65 (C) detected by western blotting. GAPDH used as a housekeeping control. Nonirradiated kidney tissues (CTL) used as controls. Expression of pNF-κBp65 and NF-κBp65 quantitated by ImageJ software and ratio of pNF-κBp65/NF-κBp65 presented (D). **p < 0.01 vs CTL; ***p < 0.001 vs CTL. (PDF 168 kb) [file 13287_2018_963_MOESM1_ESM.pdf]

Figure S2

A

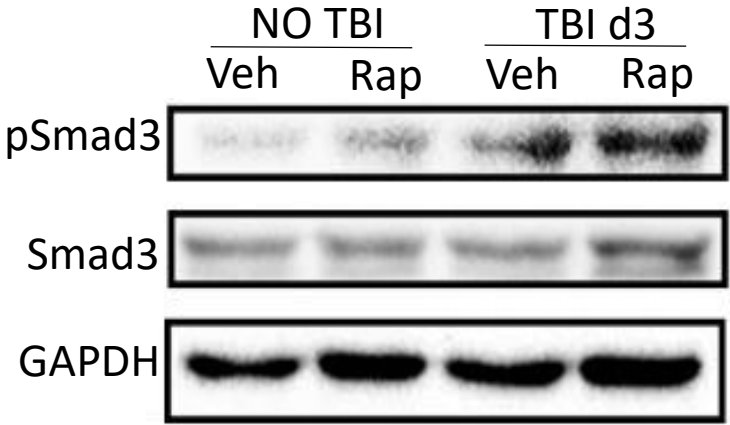

B

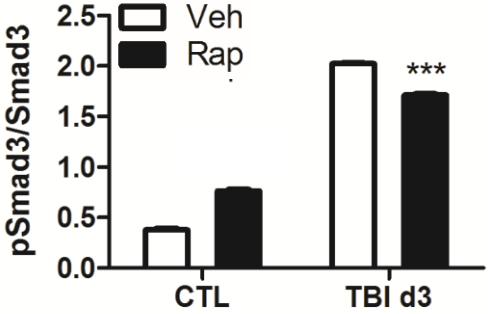

C

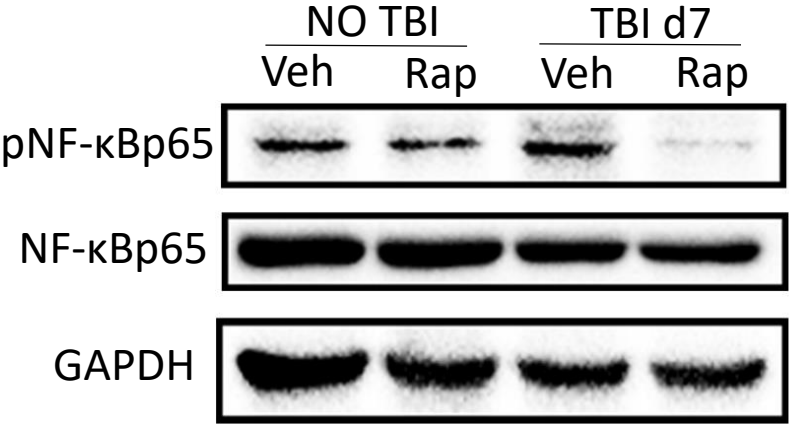

D

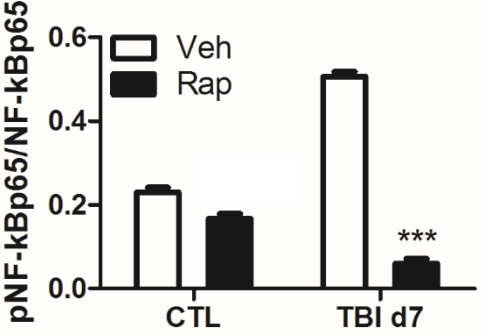

Supplement: Supplementary file 2 — Figure S2. Rapamycin treatment inhibited activation of TGF-β and NF-κB signaling induced by irradiation. C57BL/6 J mice exposed to 8.0 Gy of total body irradiation (TBI) and treated with vehicle (Veh) or rapamycin (Rap) starting at 6 h post exposure. Kidney tissues harvested at day 3 or day 7 post exposure to assess activation of TGF-β and NF-κB signaling, respectively. (A, B) Protein lysates prepared from kidney tissues at day 3 after irradiation with or without rapamycin treatment. Expression of pSmad3 and Smad3 (A) detected by western blotting. GAPDH used as a housekeeping control. Vehicle treated-kidney tissues (Veh) used as controls. Expression of pSmad3 and Smad3 quantitated by ImageJ software and ratio of pSmad3/Smad3 presented (B). (C, D) Protein lysates prepared from kidney tissues at day 7 after irradiation. Expression of pNF-κBp65 and NF-κBp65 (C) detected by western blotting. GAPDH used as a housekeeping control. Vehicle treated-kidney tissues (Veh) used as controls. Expression of pNF-κBp65 and NF-κBp65 quantitated by ImageJ software and ratio of pNF-κBp65/NF-κBp65 presented (D). ***p < 0.001 vs Veh. (PDF 163 kb) [file 13287_2018_963_MOESM2_ESM.pdf]

Figure S3

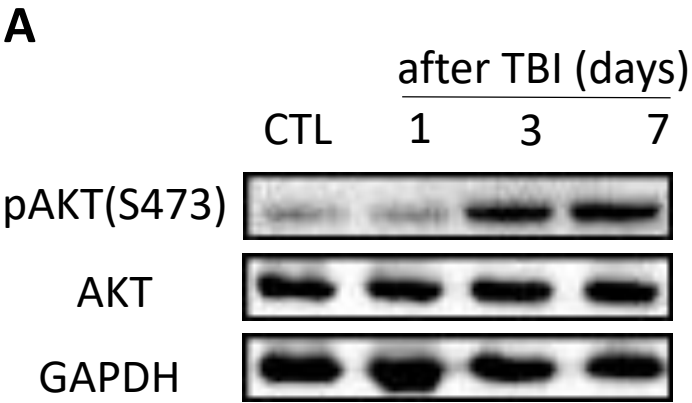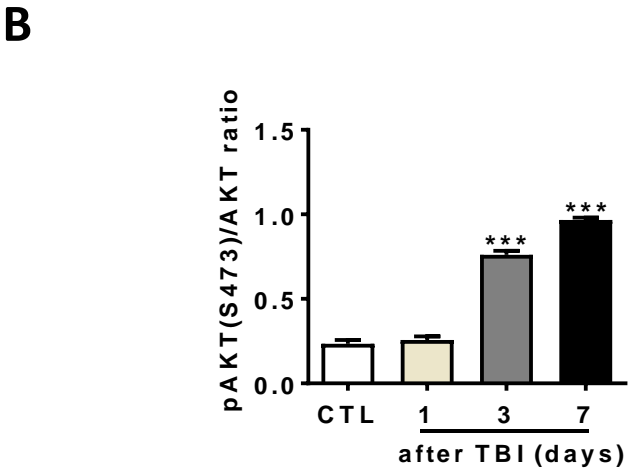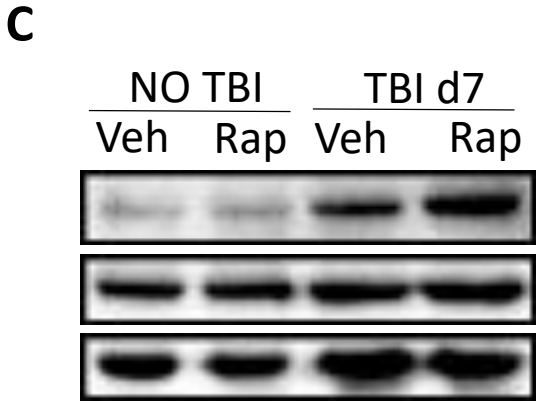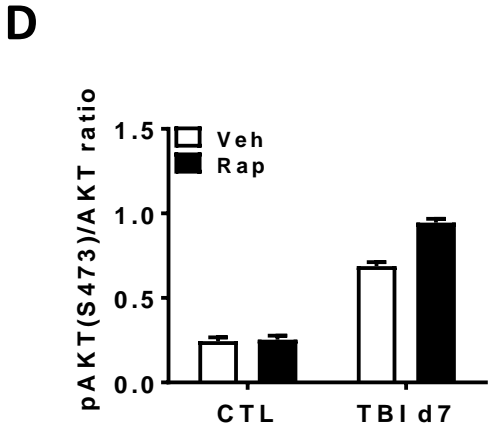

Supplement: Supplementary file 3 — Figure S3. Levels of pAkt increased by irradiation. C57BL/6 J mice exposed to 8.0 Gy of total body irradiation (TBI) and treated with vehicle (Veh) or rapamycin (Rap) starting at 6 h post exposure. Kidney tissues harvested at days 1, 3 and 7 post exposure to assess levels of phosphorylated Akt (pAkt). (A, B) Expression of pAkt upregulated post irradiation in kidney tissues. Protein lysates prepared from kidney tissues at days 1, 3 and 7 after irradiation. Expression of pAkt and Akt (A) detected by western blotting. GAPDH used as a housekeeping control. Nonirradiated kidney tissues (CTL) used as controls. Expression of pAkt and Akt quantitated by ImageJ software and ratio of pAkt/Akt presented (B). (C, D) Protein lysates prepared from kidney tissues at day 7 after irradiation. Expression of pAkt and Akt (C) detected by western blotting. GAPDH used as housekeeping control. Vehicle treated-kidney tissues (Veh) used as controls. Expression of pAkt and Akt quantitated by ImageJ software and ratio of pAkt/Akt presented (D). ***p < 0.001 vs Veh. (PDF 91 kb) [file 13287_2018_963_MOESM3_ESM.pdf]
